# Phospholipid Profiling Established by Structure‐Rich Fragments for Molecular Species Level Shotgun Analysis

**DOI:** 10.1002/rcm.70038

**Published:** 2026-01-27

**Authors:** Rong Chen, Amber H. Jannasch, Bruce R. Cooper, Jonathan H. Shannahan, Christina R. Ferreira

**Affiliations:** ^1^ Department of Chemistry Purdue University West Lafayette Indiana USA; ^2^ Metabolite Profiling Facility, Bindley Bioscience Center Purdue University West Lafayette Indiana USA; ^3^ School of Health Sciences Purdue University West Lafayette Indiana USA

**Keywords:** mass spectrometry, metabolic syndrome, phospholipids, plasmalogens, shotgun lipidomics

## Abstract

**Rationale:**

Accurate identification of phospholipid molecular species remains a major challenge in shotgun lipidomics because conventional tandem mass spectrometry typically resolves only one structural moiety at a time. This structural ambiguity limits confident lipid biomarker discovery and biological interpretation. Improving structural specificity without sacrificing analytical speed is therefore critical for lipidomics and disease‐related studies.

**Methods:**

Electrospray ionization tandem mass spectrometry was performed using direct infusion on a triple quadrupole mass spectrometer operated in multiple reaction monitoring (MRM) mode. MRMs were designed based on structure‐rich phospholipid fragments containing both the headgroup and one fatty acyl chain. Lipids were extracted from mouse liver and brain tissues and analysed without chromatographic separation, and normal‐phase LC was used for lipid headgroup confirmation only.

**Results:**

Structure‐rich MS/MS transitions enabled molecular species identification of both diacyl and ether phospholipids. 15 PUFA‐containing phospholipids were identified as candidate biomarkers differentiating healthy and metabolic syndrome mouse livers, revealing opposing regulation among structurally similar species supported by complementary fragmentation and LC evidence. In mouse brains, three ether lipid biomarkers were discovered, including plasmalogens and plasmanyl lipids, with distinct disease‐associated trends.

**Conclusion:**

This study demonstrates that structure‐rich MS/MS transitions substantially improve phospholipid structural specificity in shotgun lipidomics while maintaining high throughput. The method enables reliable identification of individual lipid species with minimal isomer interference and is readily compatible with existing workflows. This strategy offers a practical path toward more precise lipid biomarker discovery and mechanistic insight into metabolic disease.

## 1 Introduction

Phospholipids are crucial components of the cellular membrane, which facilitate complicated physiological processes occurring simultaneously in subcellular compartments [1], regulate intracellular signaling [2], and store surplus energy [3]. Altered expressions of phospholipids are closely associated with disease metabolism [4], encouraging the development of analytical tools to characterize phospholipids accurately and rapidly in complex biological matrixes. Phospholipids share a structural scaffold containing three moieties attached to the glycerol backbone [5]: the polar headgroup occupying *sn*‐3 position, as well as two fatty acyl chains occupying *sn‐*1 and *sn*‐2 positions, respectively. Each moiety can be unveiled during fragmentation of the ionized phospholipids. Specifically, the headgroup is cleaved either as a neutral or ionic species, and each fatty acyl is cleaved as a carboxylate [6]. Benefiting from the structure‐specific fragmentation, MS scans, including precursor ion scans and neutral loss scans, can be applied to target one structural moiety of lipids each time, which is the foundation of shotgun lipidomics [7] and makes it possible to profile phospholipids without chromatographic separation.

The structural diversity of phospholipids could potentially generate a large pool of isomers in biological systems [8]. Since MS scans based on conventional lipid fragments only target one structural moiety, it is challenging to resolve the remaining two moieties based on the overall configuration. For instance, MS scans targeting the headgroup could only inform the overall chain lengths and a total number of double bonds in the two fatty acyl chains, without a definitive identification of each chain. The intrinsic structural ambiguity of conventional MS scans makes it hard to elucidate all three moieties simultaneously. Novel methods are being developed to allow phospholipid characterization at deeper and more specific levels according to their structural hierarchy [9, 10, 11]: from each fatty acyl's composition to its *sn*‐position, to the double bond locations and their stereo‐configurations. In general, these methods leverage more energetic ion dissociations, multiple‐stage tandem MS, and chemical derivatization to extract more structural features than the conventional approach of single‐stage collision‐induced dissociation (CID). Ultraviolet photodissociation (UVPD) [12, 13] and electron impact excitation of ions from organics (EIEIO) [14, 15] are two novel dissociation methods that excite lipid ions at higher energies and contribute to their more extensive fragmentation to allow identification of *sn*‐positions and C=C double bond locations. MS^3^ fragmentation is another widely applied approach to revealing *sn*‐positions: CID of protonated [16], metal ion adduct [8, 17], or bicarbonate adduct [18] of phospholipids first generates a dioxolane‐type fragment [8, 16, 17] or a unique PC radical anion [18], followed by further fragmentation to yield *sn*‐specific ions. Chemical derivatization of C=C by ozonolysis [19, 20], epoxidation [21], aziridination [22], and Paternò–Büchi (PB) reaction [23, 24] forms CID‐labile products and generates diagnostic fragments to inform double bond locations. Ion mobility spectroscopy is another growing field that provides orthogonal structural information on lipid shapes and sizes [25, 26], which complements MS‐based analysis and allows more confident and comprehensive lipid identification. However, these methods are not compatible with the shotgun lipidomics workflow since they use sophisticated MS instruments equipped with novel dissociations or MS^n^ capabilities, require special sample treatment like derivatization, and often leverage the separation power from liquid chromatography.

In the current study, we have reported a new strategy of shotgun phospholipid analysis to assist in overcoming the present limitations of structure ambiguity at the molecular species level. Specifically, the strategy utilizes structure‐rich fragments of phospholipids, which are composed of the headgroup and one fatty acyl, to prepare multiple reaction monitoring (MRM) for lipid characterization. Since these fragments already contain two structural moieties of the phospholipid, it is straightforward to identify the remaining fatty acyl from the neutral loss in corresponding MRM, making them highly specific to lipid structure at the molecular species level [27]. Although these structure‐rich fragments have been observed before, we are demonstrating their first application in untargeted lipid profiling to achieve more accurate identification of lipid biomarkers. The high structural specificity of these fragments helps to offset their unfavorable detection due to the low abundance relative to major fragments. Also, we envision that detection sensitivity should be less concerning with the rapid instrument development while the structural specificity remains key to understanding important but complex biological processes at the molecular level.

As a proof‐of‐concept, we have applied the new strategy to screen phospholipids containing poly‐unsaturated fatty acids (PUFAs), which are involved in inflammation and metabolic diseases [28]. A panel of biomarkers has been discovered to vary significantly between healthy mice and those with metabolic syndrome (MetS), and the biomarker identities assigned by the novel lipid transitions have been supported using chromatographic separation. Benefiting from the specific lipid characterization, changes of individual lipid species can be accurately quantified, which reveals the diverse responses of phospholipids belonging to the same subgroup, such as those containing *ω*‐3 PUFAs. Besides the common diacyl phospholipids, we have also applied these structure‐rich transitions to characterize ether lipids, which are less abundant but have significant biological functions [29]. Consistently, three candidate biomarkers of ether lipids have been revealed with definitive moiety assignments and these biomarkers with similar structures show different variations between the two sample groups. We envision the proposed method can be easily adapted for various applications, as it requires no change of sample preparation methods or MS instruments that are currently used in conventional shotgun lipidomics.

## 2 Materials and Methods

### 2.1 Specimens and Chemicals

C57BL/6J mice (Jackson Labs, Bar Harbor, ME, USA) at 6 weeks of age were placed on either a healthy diet with 10% of kcal coming from fat (D12450B, Research Diets Inc., New Brunswick, NJ, USA), containing 51.6 mg/kg cholesterol, or a high‐fat western diet with 60% of kcal coming from fat (D12450B, Research Diets Inc.), containing 279.6 mg/kg cholesterol, to develop MetS. Biological characterization of the mouse model could be found in our other work [30], which confirms the significant increase in body weight and serum total cholesterol levels when comparing mice in the MetS group to those in the healthy group. Additionally, we have established the use of these diets to consistently produce mice with MetS in many studies [31, 32]. Mice were necropsied at 18 weeks of age to collect the liver and brain tissue, which were stored at −80°C until further use. All animal‐related procedures were conducted under the National Institutes of Health guidelines and approved by the Purdue University Animal Care and Use Committee. The mixture of lipid standards, Equisplash, was purchased from Avanti Lipids. Solvents, including methanol (MeOH) and acetonitrile (ACN), and the additive, ammonium acetate (NH_4_Ac), were purchased from Fisher Scientific.

### 2.2 Sample Preparation

Bligh–Dyer extraction was applied to both the liver and brain tissue, following the protocol reported previously [33]. Before MS analysis, the dried lipids were reconstituted in 0.5 mL MeOH and further diluted in ACN/MeOH/300 mM NH_4_Ac (3:6.65:0.35, v/v/v). The final concentration of extracts is estimated to be extractable lipids from 62.5 μg tissue per mL.

### 2.3 Instrumentation and Data Acquisition

Lipid profiling was performed via ESI using a triple quadrupole mass spectrometer (Agilent QQQ 6410), equipped with an autosampler (Agilent G1367A 1100 series) for direct flow injection. The spray voltage was 4 kV for positive ion mode and −3.5 kV for negative mode; the flow rate of solutions was 10 μL/min. During each sample injection, 10 μL of samples was delivered to the ESI source and data was acquired over 3 min. Between sample injections, the sampling needle and the transfer tubing were flushed with 100/0.1 MeOH/formic acid (FA) to avoid cross‐contamination. Besides, washing solutions were added to the sample queue to eliminate sample carryover. The collision energy used for each MRM was optimized as 25 eV. The complete list of MRMs used for profiling diacyl lipids and ether lipids is included in Figures S1–S5. Three replicates were measured for each sample.

### 2.4 Data Pretreatment and Statistics

The ion intensities of each MRM were integrated among its multiple scans over the 3 min window of data acquisition, for both samples and the blank (dilution solvent) using home‐built scripts. An intensity filter was applied to keep informative MRMs, whose signals must be 20% greater than that of the blank sample. After data filtering, each MRM intensity was normalized to the total ion counts of all MRMs in the method to obtain its relative abundance in the sample's lipid profile. A *t*‐test was further applied to discover diagnostic MRMs to distinguish healthy samples from MetS samples, with a criterion of *p* values < 0.05.

### 2.5 Chromatography Separation

Liquid chromatography was performed using a binary pump (G1312A, Agilent Technologies, Santa Clara, CA, USA), a Zorbax RX‐SIL (5 μm, 2.1 × 150 mm) column (Agilent Technologies), coupled with the MS and autosampler described above. The injection volume was 8 μL. Mobile phase A consisted of hexane/isopropanol/chloroform/water (44/43.5/10.5/2, v/v/v/v) containing 12.5 mM of ammonium formate while mobile phase B contained hexane/isopropanol/chloroform/water (34/49/10.5/6.5, v/v/v/v) with 12.5 mM of ammonium formate. The LC gradient changed as follows: (1) 0 min A/B (%) 100/0, (2) 10.5 min A/B (%) 22/78, (3) 25 min A/B (%) 22/78, (4) 25.1 min A/B (%) 100/0, and (5) 35 min A/B (%) 100/0. The flow rate was 300 μL/min, and the column was maintained at room temperature. Parameters related to MS analysis were the same as those listed above.

## 3 Results and Discussion

### 3.1 Minor Fragments of Phospholipids Benefit From Higher Structure Specificity

**FIGURE 1:**
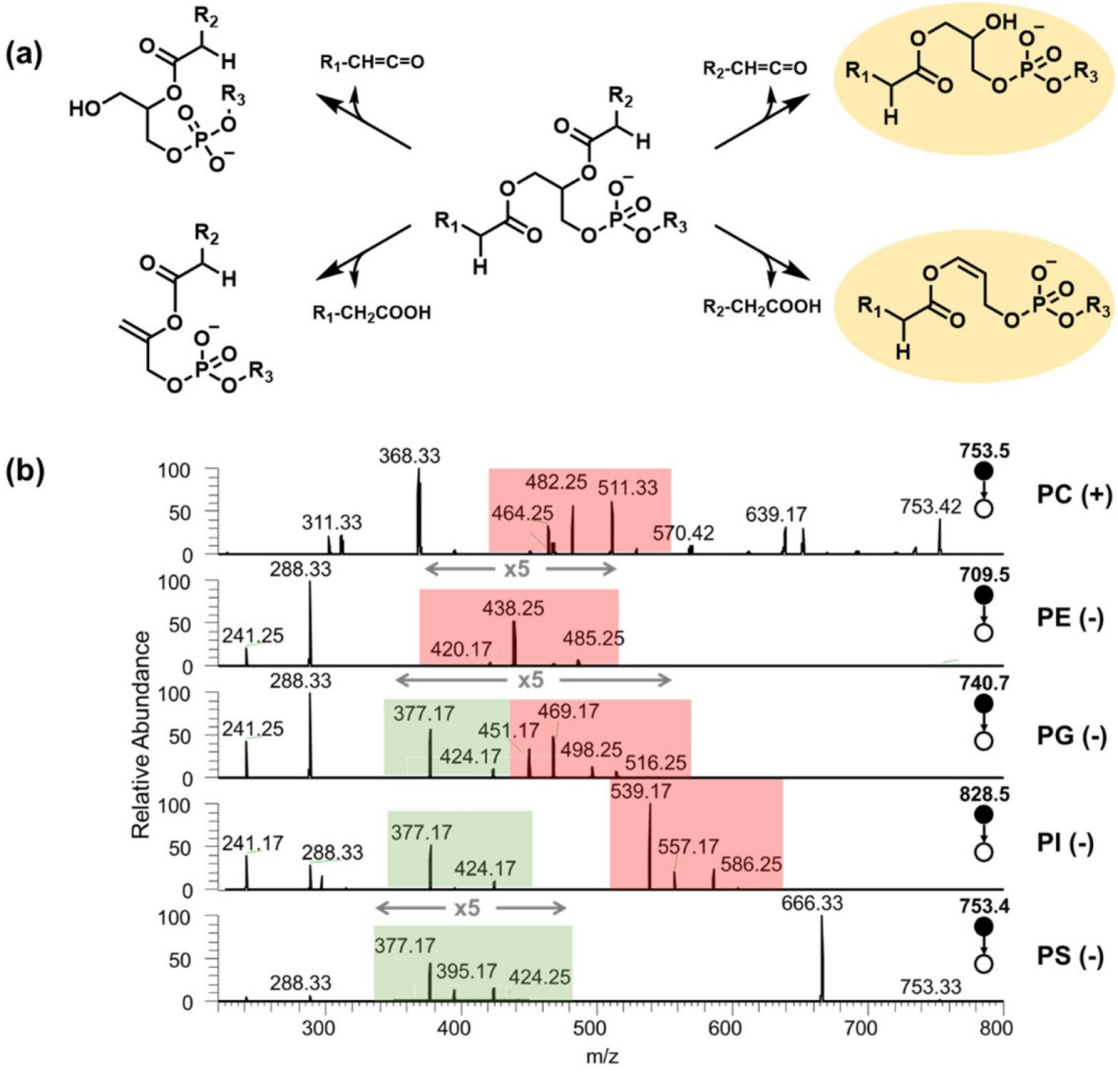
(a) Scheme shows the formation of structure‐rich phospholipid fragments via the loss of the *sn*‐1chain as either a ketene or a fatty acid, and the loss of the *sn*‐2 chain in a same manner. The highlighted fragments were observed to be favorably generated. (b) The MS/MS spectra of ionized phospholipid standards in their favorable polarities, including PC(15:0/18:1(d_7_)) detected in positive ion mode; PE(15:0/18:1(d_7_)), PG(15:0/18:1(d_7_)), PI(15:0/18:1(d_7_)), and PS(15:0/18:1(d_7_)) detected in negative mode. Peaks highlighted in red represent the structure‐rich fragments, which are commonly generated across lipid classes except PS. PS lipids tend to lose both the serine headgroup and one fatty acyl to form fragments analogous to lyso‐PAs (highlighted in green).

Minor lipid fragments, found within the lyso‐phospholipid region, are normally overlooked by conventional lipidomic approaches due to their limited intensities. However, these fragments contain richer and more specific structure information in comparison with the major fragments. During MS fragmentation of the ionized phospholipids, one fatty acyl chain is likely to be cleaved either as a neutral fatty acid or as a ketene, leaving the remaining two moieties (headgroup plus one chain) in the minor fragments (Figure 1a). Lipid transitions grounded on the minor fragments, therefore, could inform all three structural moieties of phospholipids, including the fatty acyl and the lipid headgroup indicated by the minor fragment, and the cleaved fatty acyl chain that could be simply identified from the neutral loss of the transition.

To examine the generality of the minor fragments across lipid classes, a commercial lipid mixture (EquiSplash) has been used to investigate the fragmentation spectra of multiple lipid standards. As shown in Figure 1b, the minor fragments (highlighted in red) are present in PC, PE, PG, and PI but are absent in PS. The dominant fragment at *m/z* 666 of PS suggests that PS lipids readily lose the serine headgroup during fragmentation. Consistently, the minor fragments of PS lipids are assumed to further lose their headgroups, therefore forming fragments analogous to lyso‐PA (highlighted in green). Since the fragmentation spectra of PE, PG, PI, and PS lipids were acquired in the negative polarity, their major lipid fragments representing the two fatty acyls (*m/z* 241 and 288) were also detected. Compared to these major fragments, the minor ones display limited yet detectable intensities for the PE and PG lipids, and comparatively higher relative intensities for the PI and PS lipids. For the PC lipid, its structural information is normally obtained across the two polarities with the phosphocholine headgroup detected in positive mode (*m/z* 184) and the two fatty acyls detected in negative mode (as [RCOO]^−^). The observation of minor PC fragments in its favorable positive polarity makes it possible to identify all moieties of PC lipids within one MS/MS scan. Among the four minor fragments, their relative intensities vary significantly, but a preferential loss of the *sn*‐2 chain has been observed (higher intensities for the first two peaks in the highlighted red region). One reported explanation attributes the preference to the steric hindrance effect during fragmentation [34]. In short, lipid transitions based on the minor but structure‐rich fragments could be used to screen phospholipids with improved structural specificity and to inform more comprehensive structural information of detected lipids, although having limited application on PS lipids.

### 3.2 Profile PUFA‐Containing Phospholipids in Mouse Livers

PUFAs are nutrients with significant biological functions, especially that the two subgroups, *ω*‐3 and *ω*‐6 fatty acids, coordinate opposite effects on regulating inflammation. Specifically, lipid mediators derived from *ω*‐6 fatty acids are involved in pro‐inflammatory signaling whereas those derived from *ω*‐3 fatty acids are to resolve inflammation [28]. Phospholipids containing PUFAs are the precursors and common reservoirs of PUFAs. Understanding how phospholipids regulate PUFAs could allow developing intervention and treatment strategies to address inflammation and numerous inflammation‐associated diseases. In this work, lipid transitions grounded on the minor fragments have been prepared to screen PUFA‐containing phospholipids in healthy versus MetS mouse livers, to showcase their capability of informing more specific lipid structure. Specifically, we have targeted phospholipids containing five common PUFAs, including linoleic acid (LA; C18:2, *ω*‐6), alpha linoleic acid (ALA; C18:3, *ω*‐3), arachidonic acid (AA; C20:4, *ω*‐6), eicosapentaenoic acid (EPA; C20:5, *ω*‐3), and docosahexanoic acid (DHA; C22:6, *ω*‐3). The most abundant fatty acyls in phospholipids, including C16:0, C16:1, C18:0, C18:1, C20:0, and C22:4, have been selected as the other chain while the five phospholipid classes (PC, PE, PG, PI, and PS) have all been investigated. Presumable minor fragments of these 150 potential phospholipids were calculated and the complete list can be found in Tables S1–S3.

**FIGURE 2:**
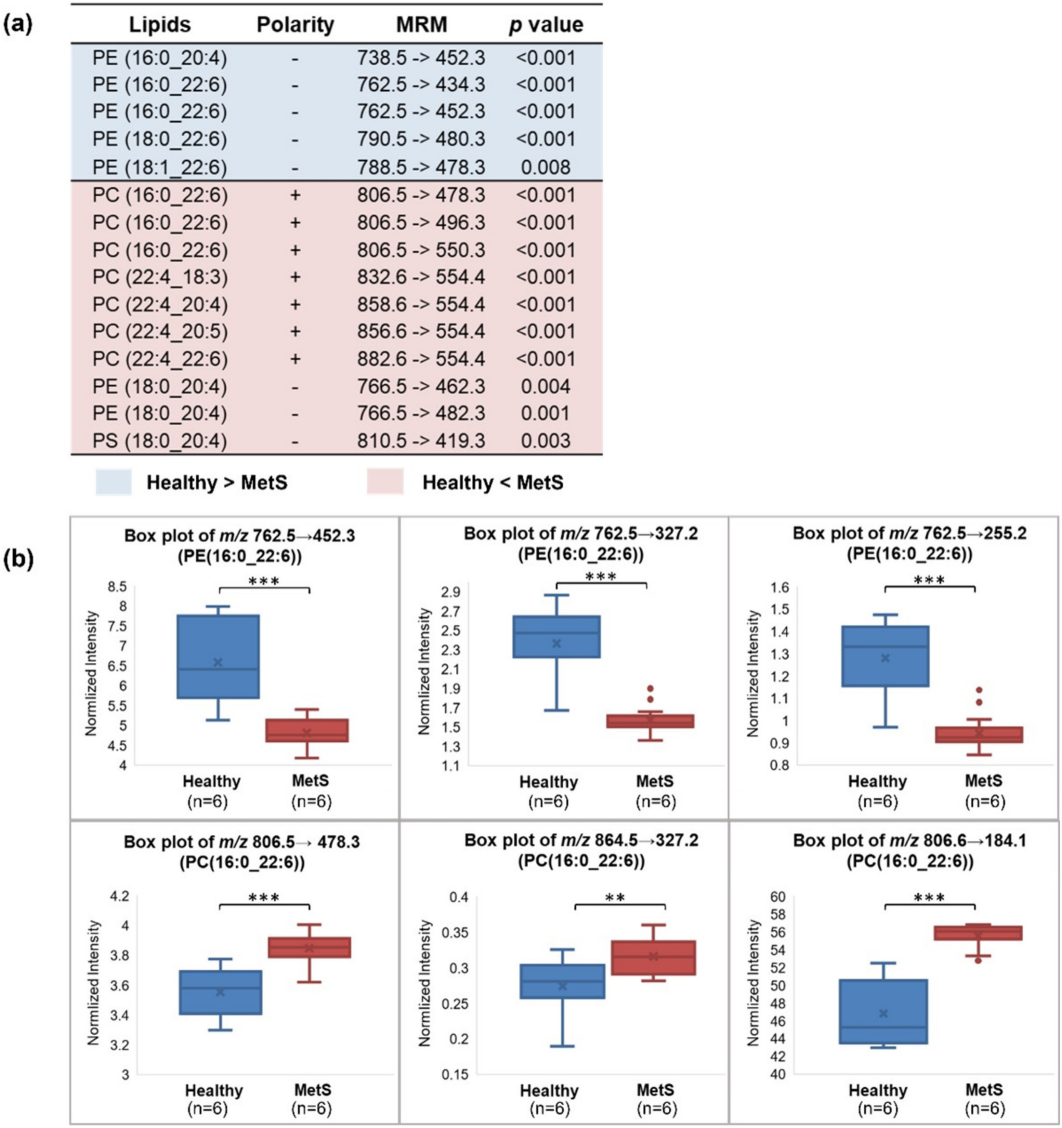
Discovery of diagnostic phospholipids by using MRMs that provide molecular species level structural identification, to distinguish healthy and MetS mice. (a) Table summarizes identified biomarkers and their diagnostic MRMs that show statistically different signals (*p* < 0.05) between the heathy versus MetS mice group. (b) Box plots of selected MRMs to show the opposite response between PC(16:0_22:6) and PE(16:0_22:6). Three asterisks indicate *p* < 0.001 and two asterisks indicate *p* < 0.01.

After screening these minor fragment‐based lipid transitions using six healthy and six MetS liver samples, data filtering, normalization and univariate statistics were applied to discover lipid transitions with significant signal variations between the two groups. Due to the intrinsically low abundance of PUFA‐containing phospholipids, only 15 diagnostic lipid transitions were found, which are summarized in Figure 2a. Most of these diagnostic transitions relate to PC and PE lipids, which agree with their dominant abundance in membrane lipids [35]. It has been observed that most PE lipids containing PUFAs are downregulated in MetS mouse livers while PC lipids containing PUFAs are upregulated. Especially, PE(16:0_22:6) and PC(16:0_22:6) demonstrated opposite responses to the disease despite their similar fatty acyl compositions (Figure 2b). This might suggest that the total abundance of DHA (22:6) in phospholipids could be too general to cover the complicated biological processes that each DHA‐containing lipid is involved in. The two phospholipids were also characterized by MRMs of their presumable major fragments, such as the PE lipid fragmenting into fatty acids (*m/z* 762.5 ➔ 255.2 in negative mode) and the PC lipid fragmenting to its headgroup (*m/z* 806.6 ➔ 184.1 in positive mode). These major transitions confirmed the downregulation of the PC biomarker and the upregulation of the PE biomarker in MetS samples (Figure 2b), consistent with the minor transitions, therefore supporting the lipid identities assigned by the structure‐rich fragments. The observed different variations of PC and PE lipids might be attributed to their distinctive effects on membrane stability, specifically PC lipids are suggested to maintain the membrane fluidity while PE lipids are to promote membrane rigidity [36]. The PC/PE ratio is crucial to liver health and a significantly altered ratio has been linked with the development of steatosis, non‐alcoholic fatty liver diseases, impaired liver regeneration, etc. [35, 37, 38].

**FIGURE 3:**
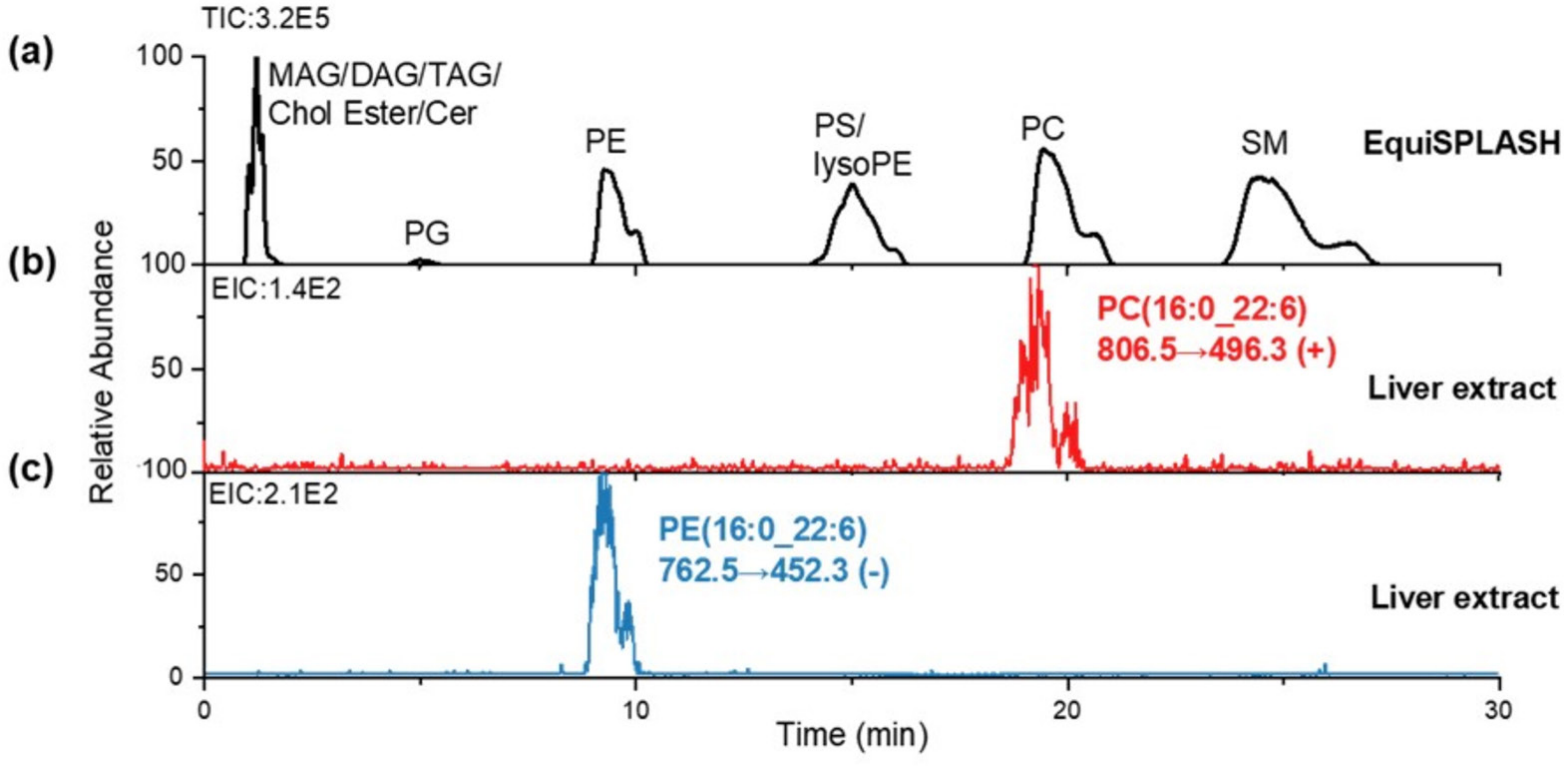
LC separation to show that lipid transitions based on structure‐rich fragments could specify the lipid classes. EquiSplash, a standard lipid mixture, has been used to indicate the retention time for each lipid class. Extracted ion chromatograms of the minor transitions are displayed to confirm their lipid classes by matching retention time.

To further confirm the identities of PE(16:0_22:6) and PC(16:0_22:6), we have characterized their lipid classes via normal phase liquid chromatography, which separates phospholipids according to their ionic headgroups. As displayed in Figure 3, the lipid species targeted by the diagnostic transition (*m/z* 806.5 ➔ 496.3) share similar retention time as the PC standard, confirming its lipid class as PC. Consistent results have been observed for the diagnostic transition targeting the assigned PE(16:0_22:6), which coeluted with the PE standard.

At the cost of a higher specificity, lipid transitions grounded on the minor fragments show a lower sensitivity than those related to major fragments. Although not well suited for the quantitation purpose, these minor transitions show promise in explorative and untargeted lipid screening. The high specificity makes these minor transitions tolerable to matrix effects; therefore, they are compatible with short or no LC separation to provide fast lipid profiling. Once tentative biomarkers are found after this first‐tier screening, their identities suggested by the minor transitions could guide the targeted quantitation using related abundant transitions to allow high sensitivity and duty cycle.

To clarify, no solid conclusion could be drawn on the biofunctions of the aforementioned phospholipids in this study, due to the limited sample size. However, it was to showcase that lipid transitions grounded on the minor but structure‐rich fragments benefit from higher structural specificity; therefore, capable of screening lipids with direct identification of headgroups and fatty acyl chains without LC separation.

### 3.3 Profile PUFA‐Containing Ether Phospholipids in Mouse Brains

Despite the predominance of diacyl phospholipids, an ether bond might attach an alkyl or alkenyl moiety to the glycerol backbone at the *sn*‐1 position, forming an ether lipid. Specifically, plasmalogens contain vinyl ether linkages at the *sn*‐1 position and plasmanyl lipids contain normal ether linkages. Ether lipids comprise approximately 20% of the total phospholipid pool in mammals but have been unveiled to affect cellular fusion, regulate cellular signaling, and function as endogenous antioxidants [29]. Considering their significant biological functions, we have explored applying our current methodology in ether lipid characterization. As ether lipids vary significantly in their distribution across different organs, which are abundant in the brain, heart, and spleen but scarce in the liver [39], we have used mouse brains for ether lipid profiling.

As an ether bond is more stable than an ester bond, we assume the alkyl/alkenyl at *sn*‐1 position would likely be retained during fragmentation while the acyl at *sn*‐2 position could be simply cleaved as its analogous diacyl lipid (scheme displayed in Figure S1). The minor fragments of ether lipids, as defined above, have been confirmed by referring to fragmentation spectra of ether lipids reported in the literature [40, 41]. Additionally, both plasmalogens and plasmanyl lipids have been observed to generate these fragments, making them versatile to target all ether lipids. Since ether lipids mostly contain choline and ethanolamine as headgroups and are enriched in PUFAs as *sn*‐2 chains, lipid transitions grounded on the structure‐rich fragments have been prepared to target potential ether lipids. Specifically, those include PC and PE ether lipids, containing common alkyls and alkenyls (C16:0, C16:1, C18:0, C18:1, C20:0, and C22:4) as the *sn*‐1 chain and common PUFAs (C18:2, C18:3, C20:4, C20:5 and C22:6) as the *sn*‐2 chain (all transitions of PC and PE ether lipids can be found in Tables S4 and S5).

**FIGURE 4:**
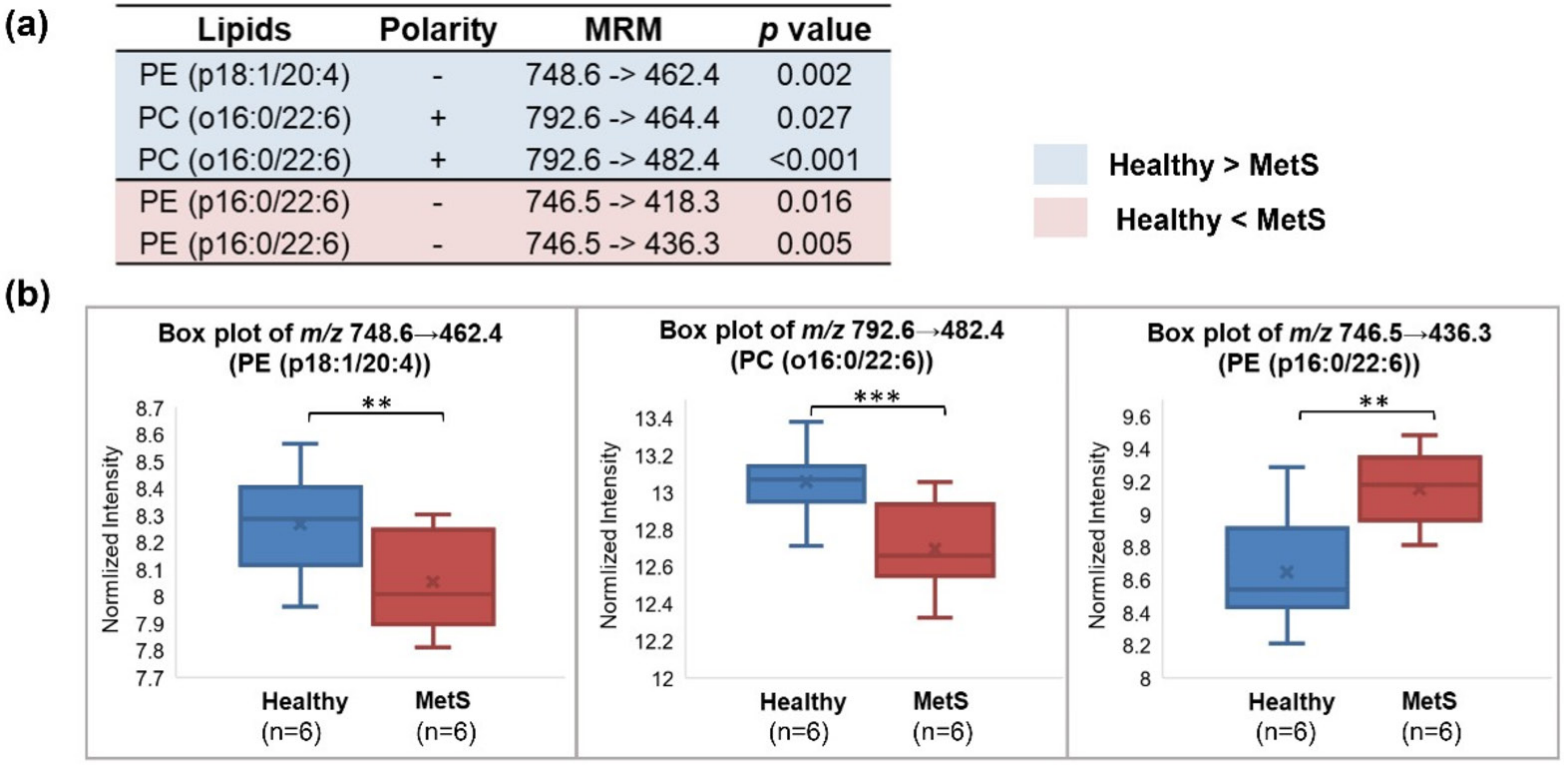
Discovery of ether lipid biomarkers by using MRMs grounded on the structure‐rich fragments, to distinguish healthy and MetS mice using the brain tissue. (a) Table summarizes identified ether lipid biomarkers and their diagnostic MRMs that show statistically different signals (*p* < 0.05) between the heathy versus MetS mice group. In the structural name, plasmanlogens are annotated by “p” while plasmanyl lipids are annotated by “o”. (b) Box plots of selected MRMs to show changes of the three identified biomarkers. Three asterisks indicate *p* < 0.001 and two asterisks indicate *p* < 0.01.

After applying these transitions to screen ether lipids in healthy (*n* = 6) and MetS (*n* = 6) mouse brains, a few were found to vary significantly between the two groups, which are summarized in Figure 4a. The five diagnostic transitions specifically pinpoint three ether lipids as potential biomarkers. The downregulation of PC(o16:0/22:6) in MetS samples (Figure 4b) may be associated with its depletion to synthesize platelet‐activating factor (PAF) via the remodeling pathway [42]. PAF is a lipid mediator that promotes inflammation and might induce multiple diseases with its enhanced production. It is noteworthy that the two identified PE plasmalogens show opposite variations based on disease status. This might echo the controversial results from previous studies which aimed to characterize plasmalogens between the control group and diseased/obese group. Some studies reported a decreased abundance of plasmalogens in disease samples due to their sacrifice in response to oxidative stresses or their depletion to release PUFAs for anti‐inflammatory effects [43], while others reported an increase of plasmalogens as a protective adaptation to mitigate the generation of oxidative species [44]. The controversy could presumably result from the distinctive function and change of individual ether lipids, making the overall characterization of the whole population not conclusive. Thus, the proposed method with high structural specificity can potentially unveil the diverse and definitive responses of individual ether lipids to metabolic diseases or inflammation.

To provide supplementary evidence to the assigned identities of these ether lipid biomarkers, we have characterized them by their diagnostic and *sn‐*specific transitions as well. For example, PE plasmalogens have been reported to form diagnostic fragments containing only *sn*‐1 or *sn*‐2 substituents following rearrangement pathways (Figure S2) [45]. And these transitions (Figure S3a,b) of the two PE biomarkers show consistent changes to those shown in Figure 4b, therefore supporting the vinyl ether and the fatty acyl composition in the plasmalogen structure. For the PC plasmanyl biomarker, its fragmentation to form the diagnostic phosphocholine (in positive mode) and the *sn*‐2 fatty acid (in negative mode) have been used as the secondary MRMs for parallel characterization. As both transitions show down‐regulation in MetS samples (Figure S3c), same as that shown in Figure 4b, the biomarker has been suggested to contain PC headgroup and arachidonic acid in its structure. Although these efforts of structural confirmation are not ideal with unavailability of standard compounds, combining multiple evidences together provides a more comprehensive view of their structure and gives us more confidence in the structural assignments.

## 4 Conclusions and Perspectives

Here, we have reported a novel lipid profiling method with improved structural specificity. To expand, the conventional lipid transitions only indicate one moiety in structure and leave the other two unresolved, while our structure‐rich transitions could elucidate two moieties at once, therefore providing straightforward identification of the last moiety. We have implemented the proposed method to characterize both diacyl phospholipids and the less abundant ether lipids between healthy and MetS mice. The high structural specificity enables the characterization of individual lipid variations with minimal isomer interference and provides richer structural information to the discovered biomarkers without further identification efforts. Therefore, this method has the potential for enriching our understanding of disease mechanisms and revealing specific functions of lipid biomarkers. Our current study has a limited sample size, which is not sufficient to produce biological conclusions. Instead, this is a proof‐of‐concept study to showcase the feasibility and advantages of the proposed method.

In this work, we have applied the method in a semi‐untargeted manner by preparing MRMs of lipids with potential interests. To maximize the screening efficiency and coverage of untargeted profiling, precursor scans grounded on the structure‐rich fragments could be applied to discover and measure precursor ions sharing the same two structural moieties. Since the novel lipid characterization is exclusively related to data acquisition during MS measurement, it does not require any modification of instruments or sample preparations, making it compatible with the current workflows of shotgun lipidomics and MRM profiling. The adaptivity of this novel methodology will hopefully promote its wide application in all aspects of phospholipid research. Lastly, the proposed method could only reveal specific structural moieties but not inform the regiospecific information, such as the *sn*‐1/*sn*‐2 position or the double bond locations.

## Author Contributions

**C.R.F.:** conceptualization, methodology, data curation, visualization, writing – original draft, writing – review and editing. **R.C.:** conceptualization, methodology, data curation, visualization, writing – original draft, writing – review and editing. **J.H.S.:** conceptualization, methodology, writing – original draft, writing – review and editing. **A.H.J.** and **B.R.C.:** resources, supervision.

## Funding

This work was supported by the National Institute of Environmental Health Sciences (R01ES033173) and the Bindley Bioscience Center (FY24/Method Devel Prog./C. Ferreira).

## Conflicts of Interest

The authors declare no conflicts of interest.

## Supporting information


**Table S1:** MRM list used for PC phospholipid profiling in the positive mode.
**Table S2:** MRM list used for PE, PI, and PG phospholipid profiling in the negative mode.
**Table S3:** MRM list used for PS phospholipid profiling in the negative mode.
**Table S4:** MRM list used for PC ether lipid profiling in the positive mode.
**Table S5:** MRM list used for PE ether lipid profiling in the negative mode.
**Figure S1:** Scheme demonstrates the formation of structure‐rich fragments of ether lipids, including the plasmalogens and plasmanyl lipids. Since the ether group stabilizes the *sn*‐1 chain, the loss of fatty acids or ketenes is expected to only occur at *sn*‐2 position.
**Figure S2:** (a) Scheme shows fragments of protonated PE plasmalogens that are specific to *sn*‐1 and *sn*‐2 chain compositions. (b) Corresponding MRMs of the two biomarkers, PE(p18:1/20:4) and PE(p16:0/22:6).
**Figure S3:** Characterizing biomarkers' changes based on disease status using their supplementary MRMs. (a, b) Characterization of the two PE plasmalogens using their diagnostic MRMs, summarized in Figure S2b. These MRMs show consistent changes of the two biomarkers with those in Figure 4b. (c) Characterization of the PC plasmanyl lipid using MRMs specific to its headgroup and *sn*‐2 fatty acid, which is down‐regulated in MetS mice as observed in Figure 4b. In the negative mode, the PC lipid ionized as the acetate adduct. Three asterisks indicate *p* < 0.001 and two asterisks indicate *p* < 0.01.

## Data Availability

The data that support the findings of this study are available upon request from the corresponding author. The data are not publicly available due to privacy or ethical restrictions.
