# Supplementary material for: Phospholipid Profiling Established by Structure‐Rich Fragments for Molecular Species Level Shotgun Analysis
Source: Rapid Commun Mass Spectrom. 2026 Jan 27;40(8):e70038. doi: 10.1002/rcm.70038 (PMC12836315; doi:10.1002/rcm.70038)
Supplement: Supplementary file 1 — Table S1: MRM list used for PC phospholipid profiling in the positive mode. Table S2: MRM list used for PE, PI, and PG phospholipid profiling in the negative mode. Table S3: MRM list used for PS phospholipid profiling in the negative mode. Table S4: MRM list used for PC ether lipid profiling in the positive mode. Table S5: MRM list used for PE ether lipid profiling in the negative mode. Figure S1: Scheme demonstrates the formation of structure‐rich fragments of ether lipids, including the plasmalogens and plasmanyl lipids. Since the ether group stabilizes the sn‐1 chain, the loss of fatty acids or ketenes is expected to only occur at sn‐2 position. Figure S2: (a) Scheme shows fragments of protonated PE plasmalogens that are specific to sn‐1 and sn‐2 chain compositions. (b) Corresponding MRMs of the two biomarkers, PE(p18:1/20:4) and PE(p16:0/22:6). Figure S3: Characterizing biomarkers' changes based on disease status using their supplementary MRMs. (a, b) Characterization of the two PE plasmalogens using their diagnostic MRMs, summarized in Figure S2b. These MRMs show consistent changes of the two biomarkers with those in Figure 4b. (c) Characterization of the PC plasmanyl lipid using MRMs specific to its headgroup and sn‐2 fatty acid, which is down‐regulated in MetS mice as observed in Figure 4b. In the negative mode, the PC lipid ionized as the acetate adduct. Three asterisks indicate p < 0.001 and two asterisks indicate p < 0.01. [file RCM-40-e70038-s001.docx]

**Supporting Information**

**Phospholipid profiling established by structure-rich fragments for molecular species level shotgun analysis.**

Rong Chen ^a^, Amber H. Jannasch ^b^, Bruce R. Cooper ^b^, Jonathan H. Shannahan ^c^, and Christina R. Ferreira ^b*^

a Department of Chemistry, Purdue University, West Lafayette, IN, 46074, USA

b Metabolite Profiling Facility, Bindley Bioscience Center, Purdue University, West Lafayette, IN, 46074, USA

c School of Health Sciences, Purdue University, West Lafayette, IN 46074, USA

* Author to whom correspondence should be addressed.

**Table S1.** MRM list used for PC phospholipid profiling in the positive mode.

| **Lipid composition** | **Precursor** | **Fragment1** | **Fragment2** | **Fragment3** | **Fragment4** |
| --- | --- | --- | --- | --- | --- |
|  | **[M+H]^+^** | **[M+H-Fatty Acid1]^+^** | **[M+H-Ketene1]^+^** | **[M+H-Fatty Acid2]^+^** | **[M+H-Ketene2]^+^** |
| PC(16:1_18:3) | 754.5 | 500.3 | 518.3 | 476.3 | 494.3 |
| PC(16:1_18:2) | 756.5 | 502.3 | 520.3 | 476.3 | 494.3 |
| PC(16:1_20:5) | 778.5 | 524.3 | 542.3 | 476.3 | 494.3 |
| PC(16:1_20:4) | 780.5 | 526.3 | 544.3 | 476.3 | 494.3 |
| PC(16:1_22:6) | 804.5 | 550.3 | 568.3 | 476.3 | 494.3 |
| PC(16:0_18:3) | 756.5 | 500.3 | 518.3 | 478.3 | 496.3 |
| PC(16:0_18:2) | 758.5 | 502.3 | 520.3 | 478.3 | 496.3 |
| PC(16:0_20:5) | 780.5 | 524.3 | 542.3 | 478.3 | 496.3 |
| PC(16:0_20:4) | 782.5 | 526.3 | 544.3 | 478.3 | 496.3 |
| PC(16:0_22:6) | 806.5 | 550.3 | 568.3 | 478.3 | 496.3 |
| PC(18:1_18:3) | 782.5 | 500.3 | 518.3 | 504.3 | 522.3 |
| PC(18:1_18:2) | 784.5 | 502.3 | 520.3 | 504.3 | 522.3 |
| PC(18:1_20:5) | 806.5 | 524.3 | 542.3 | 504.3 | 522.3 |
| PC(18:1_20:4) | 808.5 | 526.3 | 544.3 | 504.3 | 522.3 |
| PC(18:1_22:6) | 832.5 | 550.3 | 568.3 | 504.3 | 522.3 |
| PC(18:0_18:3) | 784.5 | 500.3 | 518.3 | 506.3 | 524.3 |
| PC(18:0_18:2) | 786.5 | 502.3 | 520.3 | 506.3 | 524.3 |
| PC(18:0_20:5) | 808.5 | 524.3 | 542.3 | 506.3 | 524.3 |
| PC(18:0_20:4) | 810.5 | 526.3 | 544.3 | 506.3 | 524.3 |
| PC(18:0_22:6) | 834.5 | 550.3 | 568.3 | 506.3 | 524.3 |
| PC(20:0_18:3) | 812.6 | 500.3 | 518.3 | 534.4 | 552.4 |
| PC(20:0_18:2) | 814.6 | 502.3 | 520.3 | 534.4 | 552.4 |
| PC(20:0_20:5) | 836.6 | 524.3 | 542.3 | 534.4 | 552.4 |
| PC(20:0_20:4) | 838.6 | 526.3 | 544.3 | 534.4 | 552.4 |
| PC(20:0_22:6) | 862.6 | 550.3 | 568.3 | 534.4 | 552.4 |
| PC(22:4_18:3) | 832.6 | 500.3 | 518.3 | 554.4 | 572.4 |
| PC(22:4_18:2) | 834.6 | 502.3 | 520.3 | 554.4 | 572.4 |
| PC(22:4_20:5) | 856.6 | 524.3 | 542.3 | 554.4 | 572.4 |
| PC(22:4_20:4) | 858.6 | 526.3 | 544.3 | 554.4 | 572.4 |
| PC(22:4_22:6) | 882.6 | 550.3 | 568.3 | 554.4 | 572.4 |

**Table S2.** MRM list used for PE, PI, and PG phospholipid profiling in the negative mode.

| **Lipid composition** | **Precursor** | **Fragment1** | **Fragment2** | **Fragment3** | **Fragment4** |
| --- | --- | --- | --- | --- | --- |
|  | **[M-H]^-^** | **[M-H-Fatty Acid1]^-^** | **[M-H-Ketene1]^-^** | **[M-H-Fatty Acid2]^-^** | **[M-H-Ketene2]^-^** |
| PE(16:1_18:3) | 710.5 | 456.3 | 474.3 | 432.3 | 450.3 |
| PE(16:1_18:2) | 712.5 | 458.3 | 476.3 | 432.3 | 450.3 |
| PE(16:1_20:5) | 734.5 | 480.3 | 498.3 | 432.3 | 450.3 |
| PE(16:1_20:4) | 736.5 | 482.3 | 500.3 | 432.3 | 450.3 |
| PE(16:1_22:6) | 760.5 | 506.3 | 524.3 | 432.3 | 450.3 |
| PE(16:0_18:3) | 712.5 | 456.3 | 474.3 | 434.3 | 452.3 |
| PE(16:0_18:2) | 714.5 | 458.3 | 476.3 | 434.3 | 452.3 |
| PE(16:0_20:5) | 736.5 | 480.3 | 498.3 | 434.3 | 452.3 |
| PE(16:0_20:4) | 738.5 | 482.3 | 500.3 | 434.3 | 452.3 |
| PE(16:0_22:6) | 762.5 | 506.3 | 524.3 | 434.3 | 452.3 |
| PE(18:1_18:3) | 738.5 | 456.3 | 474.3 | 460.3 | 478.3 |
| PE(18:1_18:2) | 740.5 | 458.3 | 476.3 | 460.3 | 478.3 |
| PE(18:1_20:5) | 762.5 | 480.3 | 498.3 | 460.3 | 478.3 |
| PE(18:1_20:4) | 764.5 | 482.3 | 500.3 | 460.3 | 478.3 |
| PE(18:1_22:6) | 788.5 | 506.3 | 524.3 | 460.3 | 478.3 |
| PE(18:0_18:3) | 740.5 | 456.3 | 474.3 | 462.3 | 480.3 |
| PE(18:0_18:2) | 742.5 | 458.3 | 476.3 | 462.3 | 480.3 |
| PE(18:0_20:5) | 764.5 | 480.3 | 498.3 | 462.3 | 480.3 |
| PE(18:0_20:4) | 766.5 | 482.3 | 500.3 | 462.3 | 480.3 |
| PE(18:0_22:6) | 790.5 | 506.3 | 524.3 | 462.3 | 480.3 |
| PE(20:0_18:3) | 768.6 | 456.3 | 474.3 | 490.4 | 508.4 |
| PE(20:0_18:2) | 770.6 | 458.3 | 476.3 | 490.4 | 508.4 |
| PE(20:0_20:5) | 792.6 | 480.3 | 498.3 | 490.4 | 508.4 |
| PE(20:0_20:4) | 794.6 | 482.3 | 500.3 | 490.4 | 508.4 |
| PE(20:0_22:6) | 818.6 | 506.3 | 524.3 | 490.4 | 508.4 |
| PE(22:4_18:3) | 788.6 | 456.3 | 474.3 | 510.4 | 528.4 |
| PE(22:4_18:2) | 790.6 | 458.3 | 476.3 | 510.4 | 528.4 |
| PE(22:4_20:5) | 812.6 | 480.3 | 498.3 | 510.4 | 528.4 |
| PE(22:4_20:4) | 814.6 | 482.3 | 500.3 | 510.4 | 528.4 |
| PE(22:4_22:6) | 838.6 | 506.3 | 524.3 | 510.4 | 528.4 |
| PI(16:1_18:3) | 829.5 | 575.3 | 593.3 | 551.3 | 569.3 |
| PI(16:1_18:2) | 831.5 | 577.3 | 595.3 | 551.3 | 569.3 |
| PI(16:1_20:5) | 853.5 | 599.3 | 617.3 | 551.3 | 569.3 |
| PI(16:1_20:4) | 855.5 | 601.3 | 619.3 | 551.3 | 569.3 |
| PI(16:1_22:6) | 879.5 | 625.3 | 643.3 | 551.3 | 569.3 |
| PI(16:0_18:3) | 831.5 | 575.3 | 593.3 | 553.3 | 571.3 |
| PI(16:0_18:2) | 833.5 | 577.3 | 595.3 | 553.3 | 571.3 |
| PI(16:0_20:5) | 855.5 | 599.3 | 617.3 | 553.3 | 571.3 |
| PI(16:0_20:4) | 857.5 | 601.3 | 619.3 | 553.3 | 571.3 |
| PI(16:0_22:6) | 881.5 | 625.3 | 643.3 | 553.3 | 571.3 |
| PI(18:1_18:3) | 857.5 | 575.3 | 593.3 | 579.3 | 597.3 |
| PI(18:1_18:2) | 859.5 | 577.3 | 595.3 | 579.3 | 597.3 |
| PI(18:1_20:5) | 881.5 | 599.3 | 617.3 | 579.3 | 597.3 |
| PI(18:1_20:4) | 883.5 | 601.3 | 619.3 | 579.3 | 597.3 |
| PI(18:1_22:6) | 907.5 | 625.3 | 643.3 | 579.3 | 597.3 |
| PI(18:0_18:3) | 859.5 | 575.3 | 593.3 | 581.3 | 599.3 |
| PI(18:0_18:2) | 861.5 | 577.3 | 595.3 | 581.3 | 599.3 |
| PI(18:0_20:5) | 883.5 | 599.3 | 617.3 | 581.3 | 599.3 |
| PI(18:0_20:4) | 885.5 | 601.3 | 619.3 | 581.3 | 599.3 |
| PI(18:0_22:6) | 909.5 | 625.3 | 643.3 | 581.3 | 599.3 |
| PI(20:0_18:3) | 887.6 | 575.3 | 593.3 | 609.4 | 627.4 |
| PI(20:0_18:2) | 889.6 | 577.3 | 595.3 | 609.4 | 627.4 |
| PI(20:0_20:5) | 911.6 | 599.3 | 617.3 | 609.4 | 627.4 |
| PI(20:0_20:4) | 913.6 | 601.3 | 619.3 | 609.4 | 627.4 |
| PI(20:0_22:6) | 937.6 | 625.3 | 643.3 | 609.4 | 627.4 |
| PI(22:4_18:3) | 907.6 | 575.3 | 593.3 | 629.4 | 647.4 |
| PI(22:4_18:2) | 909.6 | 577.3 | 595.3 | 629.4 | 647.4 |
| PI(22:4_20:5) | 931.6 | 599.3 | 617.3 | 629.4 | 647.4 |
| PI(22:4_20:4) | 933.6 | 601.3 | 619.3 | 629.4 | 647.4 |
| PI(22:4_22:6) | 957.6 | 625.3 | 643.3 | 629.4 | 647.4 |
| PG(16:1_18:3) | 741.5 | 487.3 | 505.3 | 463.3 | 481.3 |
| PG(16:1_18:2) | 743.5 | 489.3 | 507.3 | 463.3 | 481.3 |
| PG(16:1_20:5) | 765.5 | 511.3 | 529.3 | 463.3 | 481.3 |
| PG(16:1_20:4) | 767.5 | 513.3 | 531.3 | 463.3 | 481.3 |
| PG(16:1_22:6) | 791.5 | 537.5 | 555.3 | 463.3 | 481.3 |
| PG(16:0_18:3) | 743.5 | 487.3 | 505.3 | 465.3 | 483.3 |
| PG(16:0_18:2) | 745.5 | 489.3 | 507.3 | 465.3 | 483.3 |
| PG(16:0_20:5) | 767.5 | 511.3 | 529.3 | 465.3 | 483.3 |
| PG(16:0_20:4) | 769.5 | 513.3 | 531.3 | 465.3 | 483.3 |
| PG(16:0_22:6) | 793.5 | 537.5 | 555.3 | 465.3 | 483.3 |
| PG(18:1_18:3) | 769.5 | 487.3 | 505.3 | 491.3 | 509.3 |
| PG(18:1_18:2) | 771.5 | 489.3 | 507.3 | 491.3 | 509.3 |
| PG(18:1_20:5) | 793.5 | 511.3 | 529.3 | 491.3 | 509.3 |
| PG(18:1_20:4) | 795.5 | 513.3 | 531.3 | 491.3 | 509.3 |
| PG(18:1_22:6) | 819.5 | 537.5 | 555.3 | 491.3 | 509.3 |
| PG(18:0_18:3) | 771.5 | 487.3 | 505.3 | 493.3 | 511.3 |
| PG(18:0_18:2) | 773.5 | 489.3 | 507.3 | 493.3 | 511.3 |
| PG(18:0_20:5) | 795.5 | 511.3 | 529.3 | 493.3 | 511.3 |
| PG(18:0_20:4) | 797.5 | 513.3 | 531.3 | 493.3 | 511.3 |
| PG(18:0_22:6) | 821.5 | 537.5 | 555.3 | 493.3 | 511.3 |
| PG(20:0_18:3) | 799.6 | 487.3 | 505.3 | 521.4 | 539.4 |
| PG(20:0_18:2) | 801.6 | 489.3 | 507.3 | 521.4 | 539.4 |
| PG(20:0_20:5) | 823.6 | 511.3 | 529.3 | 521.4 | 539.4 |
| PG(20:0_20:4) | 825.6 | 513.3 | 531.3 | 521.4 | 539.4 |
| PG(20:0_22:6) | 849.6 | 537.5 | 555.3 | 521.4 | 539.4 |
| PG(22:4_18:3) | 819.6 | 487.3 | 505.3 | 541.4 | 559.4 |
| PG(22:4_18:2) | 821.6 | 489.3 | 507.3 | 541.4 | 559.4 |
| PG(22:4_20:5) | 843.6 | 511.3 | 529.3 | 541.4 | 559.4 |
| PG(22:4_20:4) | 845.6 | 513.3 | 531.3 | 541.4 | 559.4 |
| PG(22:4_22:6) | 869.6 | 537.5 | 555.3 | 541.4 | 559.4 |

**Table S3.** MRM list used for PS phospholipid profiling in the negative mode.

| **Lipid composition** | **Precursor** | **Fragment1** | **Fragment2** | **Fragment3** | **Fragment4** |
| --- | --- | --- | --- | --- | --- |
|  | **[M-H]^-^** | **[M-H-Serine-Fatty Acid1]^-^** | **[M-H-Serine-Ketene1]^-^** | **[M-H-Serine-Fatty Acid2]^-^** | **[M-H-Serine-Ketene2]^-^** |
| PS(16:1_18:3) | 754.5 | 413.3 | 431.3 | 389.3 | 407.3 |
| PS(16:1_18:2) | 756.5 | 415.3 | 433.3 | 389.3 | 407.3 |
| PS(16:1_20:5) | 778.5 | 437.3 | 455.3 | 389.3 | 407.3 |
| PS(16:1_20:4) | 780.5 | 439.3 | 457.3 | 389.3 | 407.3 |
| PS(16:1_22:6) | 804.5 | 463.3 | 481.3 | 389.3 | 407.3 |
| PS(16:0_18:3) | 756.5 | 413.3 | 431.3 | 391.3 | 409.3 |
| PS(16:0_18:2) | 758.5 | 415.3 | 433.3 | 391.3 | 409.3 |
| PS(16:0_20:5) | 780.5 | 437.3 | 455.3 | 391.3 | 409.3 |
| PS(16:0_20:4) | 782.5 | 439.3 | 457.3 | 391.3 | 409.3 |
| PS(16:0_22:6) | 806.5 | 463.3 | 481.3 | 391.3 | 409.3 |
| PS(18:1_18:3) | 782.5 | 413.3 | 431.3 | 417.3 | 435.3 |
| PS(18:1_18:2) | 784.5 | 415.3 | 433.3 | 417.3 | 435.3 |
| PS(18:1_20:5) | 806.5 | 437.3 | 455.3 | 417.3 | 435.3 |
| PS(18:1_20:4) | 808.5 | 439.3 | 457.3 | 417.3 | 435.3 |
| PS(18:1_22:6) | 832.5 | 463.3 | 481.3 | 417.3 | 435.3 |
| PS(18:0_18:3) | 784.5 | 413.3 | 431.3 | 419.3 | 437.3 |
| PS(18:0_18:2) | 786.5 | 415.3 | 433.3 | 419.3 | 437.3 |
| PS(18:0_20:5) | 808.5 | 437.3 | 455.3 | 419.3 | 437.3 |
| PS(18:0_20:4) | 810.5 | 439.3 | 457.3 | 419.3 | 437.3 |
| PS(18:0_22:6) | 834.5 | 463.3 | 481.3 | 419.3 | 437.3 |
| PS(20:0_18:3) | 812.6 | 413.3 | 431.3 | 447.4 | 465.4 |
| PS(20:0_18:2) | 814.6 | 415.3 | 433.3 | 447.4 | 465.4 |
| PS(20:0_20:5) | 836.6 | 437.3 | 455.3 | 447.4 | 465.4 |
| PS(20:0_20:4) | 838.6 | 439.3 | 457.3 | 447.4 | 465.4 |
| PS(20:0_22:6) | 862.6 | 463.3 | 481.3 | 447.4 | 465.4 |
| PS(22:4_18:3) | 832.6 | 413.3 | 431.3 | 467.4 | 485.4 |
| PS(22:4_18:2) | 834.6 | 415.3 | 433.3 | 467.4 | 485.4 |
| PS(22:4_20:5) | 856.6 | 437.3 | 455.3 | 467.4 | 485.4 |
| PS(22:4_20:4) | 858.6 | 439.3 | 457.3 | 467.4 | 485.4 |
| PS(22:4_22:6) | 882.6 | 463.3 | 481.3 | 467.4 | 485.4 |

* To note, the minor fragments of PS lipids are analogous to lyso-PA lipids since they have lost the serine headgroup.

**Table S4.** MRM list used for PC ether lipid profiling in the positive mode.

| **Lipid composition** | **Precursor** | **Fragment1** | **Fragment2** | **Lipid composition** | **Precursor** | **Fragment1** | **Fragment2** |
| --- | --- | --- | --- | --- | --- | --- | --- |
|  | **[M+H]^+^** | **[M+H-Fatty Acid]^+^** | **[M+H-Ketene]^+^** |  | **[M+H]^+^** | **[M+H-Fatty Acid]^+^** | **[M+H-Ketene]^+^** |
| PC(p16:1/18:3) | 738.5 | 460.3 | 478.3 | PC(o16:1/18:3) | 740.5 | 462.3 | 480.3 |
| PC(p16:1/18:2) | 740.5 | 460.3 | 478.3 | PC(o16:1/18:2) | 742.5 | 462.3 | 480.3 |
| PC(p16:1/20:5) | 762.5 | 460.3 | 478.3 | PC(o16:1/20:5) | 764.5 | 462.3 | 480.3 |
| PC(p16:1/20:4) | 764.5 | 460.3 | 478.3 | PC(o16:1/20:4) | 766.5 | 462.3 | 480.3 |
| PC(p16:1/22:6) | 788.5 | 460.3 | 478.3 | PC(o16:1/22:6) | 790.5 | 462.3 | 480.3 |
| PC(p16:0/18:3) | 740.5 | 462.3 | 480.3 | PC(o16:0/18:3) | 742.5 | 464.4 | 482.4 |
| PC(p16:0/18:2) | 742.5 | 462.3 | 480.3 | PC(o16:0/18:2) | 744.5 | 464.4 | 482.4 |
| PC(p16:0/20:5) | 764.5 | 462.3 | 480.3 | PC(o16:0/20:5) | 766.5 | 464.4 | 482.4 |
| PC(p16:0/20:4) | 766.5 | 462.3 | 480.3 | PC(o16:0/20:4) | 768.6 | 464.4 | 482.4 |
| PC(p16:0/22:6) | 790.5 | 462.3 | 480.3 | PC(o16:0/22:6) | 792.6 | 464.4 | 482.4 |
| PC(p18:1/18:3) | 766.6 | 488.4 | 506.4 | PC(o18:1/18:3) | 768.6 | 490.4 | 508.4 |
| PC(p18:1/18:2) | 768.6 | 488.4 | 506.4 | PC(o18:1/18:2) | 770.6 | 490.4 | 508.4 |
| PC(p18:1/20:5) | 790.6 | 488.4 | 506.4 | PC(o18:1/20:5) | 792.6 | 490.4 | 508.4 |
| PC(p18:1/20:4) | 792.6 | 488.4 | 506.4 | PC(o18:1/20:4) | 794.6 | 490.4 | 508.4 |
| PC(p18:1/22:6) | 816.6 | 488.4 | 506.4 | PC(o18:1/22:6) | 818.6 | 490.4 | 508.4 |
| PC(p18:0/18:3) | 768.6 | 490.4 | 508.4 | PC(o18:0/18:3) | 770.6 | 492.4 | 510.4 |
| PC(p18:0/18:2) | 770.6 | 490.4 | 508.4 | PC(o18:0/18:2) | 772.6 | 492.4 | 510.4 |
| PC(p18:0/20:5) | 792.6 | 490.4 | 508.4 | PC(o18:0/20:5) | 794.6 | 492.4 | 510.4 |
| PC(p18:0/20:4) | 794.6 | 490.4 | 508.4 | PC(o18:0/20:4) | 796.6 | 492.4 | 510.4 |
| PC(p18:0/22:6) | 818.6 | 490.4 | 508.4 | PC(o18:0/22:6) | 820.6 | 492.4 | 510.4 |
| PC(p20:0/18:3) | 796.6 | 518.4 | 536.4 | PC(o20:0/18:3) | 798.6 | 520.4 | 538.4 |
| PC(p20:0/18:2) | 798.6 | 518.4 | 536.4 | PC(o20:0/18:2) | 800.6 | 520.4 | 538.4 |
| PC(p20:0/20:5) | 820.6 | 518.4 | 536.4 | PC(o20:0/20:5) | 822.6 | 520.4 | 538.4 |
| PC(p20:0/20:4) | 822.6 | 518.4 | 536.4 | PC(o20:0/20:4) | 824.6 | 520.4 | 538.4 |
| PC(p20:0/22:6) | 846.6 | 518.4 | 536.4 | PC(o20:0/22:6) | 848.6 | 520.4 | 538.4 |
| PC(p22:4/18:3) | 824.6 | 546.4 | 564.4 | PC(o22:4/18:3) | 826.6 | 548.4 | 566.4 |
| PC(p22:4/18:2) | 826.6 | 546.4 | 564.4 | PC(o22:4/18:2) | 828.6 | 548.4 | 566.4 |
| PC(p22:4/20:5) | 848.6 | 546.4 | 564.4 | PC(o22:4/20:5) | 850.6 | 548.4 | 566.4 |
| PC(p22:4/20:4) | 850.6 | 546.4 | 564.4 | PC(o22:4/20:4) | 852.6 | 548.4 | 566.4 |
| PC(p22:4/22:6) | 874.6 | 546.4 | 564.4 | PC(o22:4/22:6) | 876.6 | 548.4 | 566.4 |

* As the proposed method could not specify double bond position within a fatty acyl chain, isomers like PC(p16:0_18:3) and PC(o16:1_18:3) share the same structure-rich fragments. **Table S5.** MRM list used for PE ether lipid profiling in the negative mode.

| **Lipid composition** | **Precursor** | **Fragment1** | **Fragment2** | **Lipid composition** | **Precursor** | **Fragment1** | **Fragment2** |
| --- | --- | --- | --- | --- | --- | --- | --- |
|  | **[M-H]^-^** | **[M-H-Fatty Acid]^-^** | **[M-H-Ketene]^-^** |  | **[M-H]^-^** | **[M-H-Fatty Acid]^-^** | **[M-H-Ketene]^-^** |
| PE(p16:1/18:3) | 694.5 | 416.3 | 434.3 | PE(o16:1/18:3) | 696.5 | 418.3 | 436.3 |
| PE(p16:1/18:2) | 696.5 | 416.3 | 434.3 | PE(o16:1/18:2) | 698.5 | 418.3 | 436.3 |
| PE(p16:1/20:5) | 718.5 | 416.3 | 434.3 | PE(o16:1/20:5) | 720.5 | 418.3 | 436.3 |
| PE(p16:1/20:4) | 720.5 | 416.3 | 434.3 | PE(o16:1/20:4) | 722.5 | 418.3 | 436.3 |
| PE(p16:1/22:6) | 744.5 | 416.3 | 434.3 | PE(o16:1/22:6) | 746.5 | 418.3 | 436.3 |
| PE(p16:0/18:3) | 696.5 | 418.3 | 436.3 | PE(o16:0/18:3) | 698.6 | 420.4 | 438.4 |
| PE(p16:0/18:2) | 698.5 | 418.3 | 436.3 | PE(o16:0/18:2) | 700.6 | 420.4 | 438.4 |
| PE(p16:0/20:5) | 720.5 | 418.3 | 436.3 | PE(o16:0/20:5) | 722.6 | 420.4 | 438.4 |
| PE(p16:0/20:4) | 722.5 | 418.3 | 436.3 | PE(o16:0/20:4) | 724.6 | 420.4 | 438.4 |
| PE(p16:0/22:6) | 746.5 | 418.3 | 436.3 | PE(o16:0/22:6) | 748.6 | 420.4 | 438.4 |
| PE(p18:1/18:3) | 722.6 | 444.4 | 462.4 | PE(o18:1/18:3) | 724.6 | 446.4 | 464.4 |
| PE(p18:1/18:2) | 724.6 | 444.4 | 462.4 | PE(o18:1/18:2) | 726.6 | 446.4 | 464.4 |
| PE(p18:1/20:5) | 746.6 | 444.4 | 462.4 | PE(o18:1/20:5) | 748.6 | 446.4 | 464.4 |
| PE(p18:1/20:4) | 748.6 | 444.4 | 462.4 | PE(o18:1/20:4) | 750.6 | 446.4 | 464.4 |
| PE(p18:1/22:6) | 772.6 | 444.4 | 462.4 | PE(o18:1/22:6) | 774.6 | 446.4 | 464.4 |
| PE(p18:0/18:3) | 724.6 | 446.4 | 464.4 | PE(o18:0/18:3) | 726.6 | 448.4 | 466.4 |
| PE(p18:0/18:2) | 726.6 | 446.4 | 464.4 | PE(o18:0/18:2) | 728.6 | 448.4 | 466.4 |
| PE(p18:0/20:5) | 748.6 | 446.4 | 464.4 | PE(o18:0/20:5) | 750.6 | 448.4 | 466.4 |
| PE(p18:0/20:4) | 750.6 | 446.4 | 464.4 | PE(o18:0/20:4) | 752.6 | 448.4 | 466.4 |
| PE(p18:0/22:6) | 774.6 | 446.4 | 464.4 | PE(o18:0/22:6) | 776.6 | 448.4 | 466.4 |
| PE(p20:0/18:3) | 752.6 | 474.4 | 492.4 | PE(o20:0/18:3) | 754.6 | 476.4 | 494.4 |
| PE(p20:0/18:2) | 754.6 | 474.4 | 492.4 | PE(o20:0/18:2) | 756.6 | 476.4 | 494.4 |
| PE(p20:0/20:5) | 776.6 | 474.4 | 492.4 | PE(o20:0/20:5) | 778.6 | 476.4 | 494.4 |
| PE(p20:0/20:4) | 778.6 | 474.4 | 492.4 | PE(o20:0/20:4) | 780.6 | 476.4 | 494.4 |
| PE(p20:0/22:6) | 802.6 | 474.4 | 492.4 | PE(o20:0/22:6) | 804.6 | 476.4 | 494.4 |
| PE(p22:4/18:3) | 780.6 | 502.4 | 520.4 | PE(o22:4/18:3) | 782.6 | 504.4 | 522.4 |
| PE(p22:4/18:2) | 782.6 | 502.4 | 520.4 | PE(o22:4/18:2) | 784.6 | 504.4 | 522.4 |
| PE(p22:4/20:5) | 804.6 | 502.4 | 520.4 | PE(o22:4/20:5) | 806.6 | 504.4 | 522.4 |
| PE(p22:4/20:4) | 806.6 | 502.4 | 520.4 | PE(o22:4/20:4) | 808.6 | 504.4 | 522.4 |
| PE(p22:4/22:6) | 830.6 | 502.4 | 520.4 | PE(o22:4/22:6) | 832.6 | 504.4 | 522.4 |

* As the proposed method could not specify double bond position within a fatty acyl chain, isomers like PE(p16:0_18:3) and PE(o16:1_18:3) share the same structure-rich fragments.

**Figure S1.** Scheme demonstrates the formation of structure-rich fragments of ether lipids, including the plasmalogens and plasmanyl lipids. Since the ether group stabilizes the *sn*-1 chain, the loss of fatty acids or ketenes is expected to only occur at *sn*-2 position.


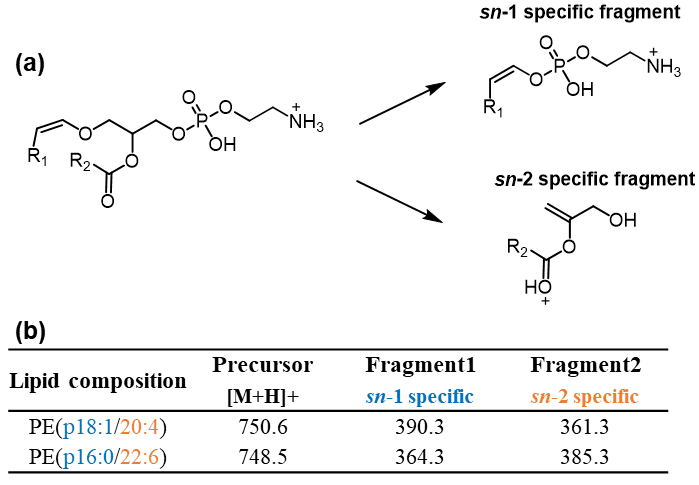


**Figure S2.** **(a)** Scheme shows fragments of protonated PE plasmalogens that are specific to *sn*-1 and *sn*-2 chain compositions. **(b)** Corresponding MRMs of the two biomarkers, PE(p18:1/20:4) and PE(p16:0/22:6).


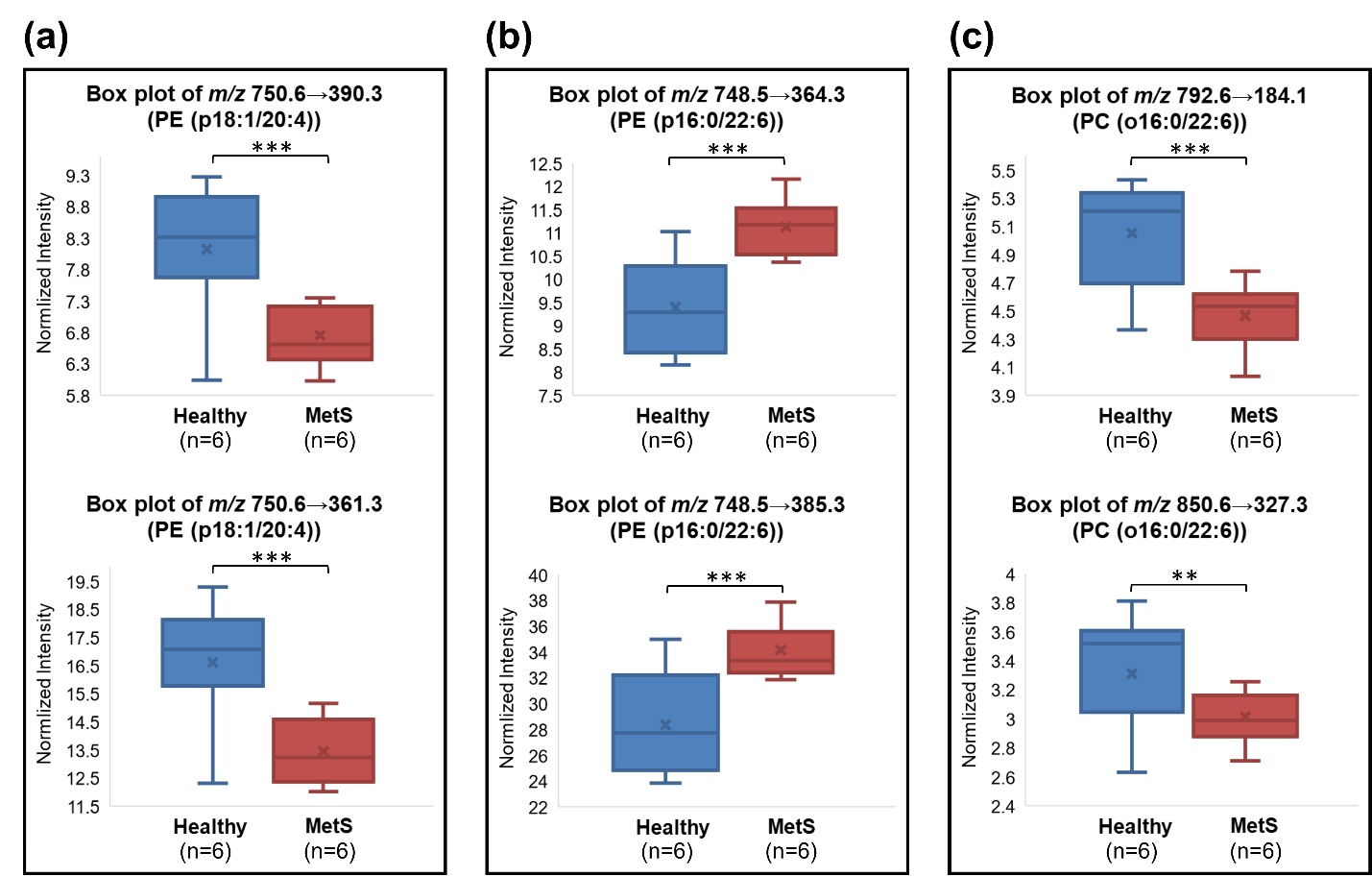


**Figure S3.** Characterizing biomarkers’ changes based on disease status using their supplementary MRMs. **(a-b)** Characterization of the two PE plasmalogens using their diagnostic MRMs, summarized in Fig S2b. These MRMs show consistent changes of the two biomarkers with those in Fig 4b. **(c)** Characterization of the PC plasmanyl lipid using MRMs specific to its headgroup and *sn*-2 fatty acid, which is down-regulated in MetS mice as observed in Fig 4b. In the negative mode, the PC lipid ionized as the acetate adduct. Three asterisks indicate *p*<0.001 and two asterisks indicate *p*<0.01.
